# Supplementary material for: Catch, bycatch and discards of the Galapagos Marine Reserve small-scale handline fishery
Source: PeerJ. 2015 Jun 9;3:e995. doi: 10.7717/peerj.995 (PMC4465951; doi:10.7717/peerj.995)
Supplement: Annex S1 — Fitting parameters (a and b) of the length-weight relationship and the number of individuals measured (n). [file peerj-03-995-s001.docx]

| **Species** | **a** | **b** | **n** |
| --- | --- | --- | --- |
| *Hemilutjanus macrophthalmos* | *0.07* | *2.54* | *95* |
| *Pontinus clemensi* | *0.01* | *3.21* | *120* |
| *Semicossyphus darwini* | *0.11* | *2.50* | *96* |
