# Supplementary material for: Catch, bycatch and discards of the Galapagos Marine Reserve small-scale handline fishery
Source: PeerJ. 2015 Jun 9;3:e995. doi: 10.7717/peerj.995 (PMC4465951; doi:10.7717/peerj.995)
Supplement: Annex S2 — Shows the species that were mentioned once by interviewees as well as the reasons to not land these species. [file peerj-03-995-s002.docx]

| **Species** | **Not marketable species** | **Not marketable size** |
| --- | --- | --- |
| *Caulolatilus affinis* | 1 |  |
| *Caranx caballus* | 1 |  |
| *Dermatolepis dermatolepis* |  | 1 |
| *Epinephelus cifuentesi* |  | 1 |
| *Eucinostomus dowii* | 1 |  |
| *Euthynnus lineatus* | 1 |  |
| *Haemulon sexfasciatum* | 1 |  |
| *Lutjanus sp.* |  | 1 |
| *Mugil galapagensis* |  | 1 |
| *Murraena sp.* | 1 |  |
| *Mycteroperca olfax** |  | 1 |
| *Myrichthys tigrinus* | 1 |  |
| *Paralabrax albomaculatus** |  | 1 |
| *Semicossyphus darwini* | 1 |  |
| *Sphyraena idiastes* | 1 |  |
| *Thunnus albacares* |  | 1 |
| *Xenichthys sp.* | 1 |  |
